# Effects of the Water-Based Foaming Process on the Basic and Rheological Properties of Bitumen 70/100

**DOI:** 10.3390/ma14112803

**Published:** 2021-05-25

**Authors:** Marek Iwański, Anna Chomicz-Kowalska, Grzegorz Mazurek, Przemysław Buczyński, Małgorzata Cholewińska, Mateusz M. Iwański, Krzysztof Maciejewski, Piotr Ramiączek

**Affiliations:** Department of Civil Engineering and Architecture, Kielce University of Technology, Al. Tysiąclecia Państwa Polskiego 7, 25-314 Kielce, Poland; a.kowalska@tu.kielce.pl (A.C.-K.); gmazurek@tu.kielce.pl (G.M.); p.buczynski@tu.kielce.pl (P.B.); m.cholewinska@tu.kielce.pl (M.C.); matiwanski@tu.kielce.pl (M.M.I.); kmaciejewski@tu.kielce.pl (K.M.); piotrr@tu.kielce.pl (P.R.)

**Keywords:** water-foamed bitumen, fundamental properties, rheology, low-temperature properties, 2S2P1D model

## Abstract

The process of water-based foaming of bitumen produces binders that can be incorporated in cold recycled asphalt mixes and pavement upper structural layers made of half-warm mix asphalt prepared at 100–130 °C. During the foaming process, cold water and air act on hot bitumen (160–170 °C), which results in the explosive vaporization of water leading to changes in the binder structure. The impact of foaming on the properties of bitumen 70/100 was evaluated by investigating the binder characteristics before and after foaming. Determination of two foaming parameters, maximum expansion and half-life, was followed by measurements of penetration at 25 °C, softening point, Fraass breaking point, and dynamic viscosity at 60, 90, and 135 °C. Rheological and low-temperature tests were also performed before and after foaming bitumen 70/100. The Bending Beam Rheometer method was applied to determine the low temperature stiffness modulus. A DHR-2 rheometer was used to determine the dynamic modulus and phase angle of the tested binder. The Black and master curves before and after foaming were plotted in the 2S2P1D model and the model parameters were analysed. Analysis of the test results confirmed the effects of the foaming process on the basic, low-temperature, and rheological characteristics of the bitumen.

## 1. Introduction

Dynamic development of water-foamed bitumen technology started at the turn of the 21st century with the research led by prof. K. J. Jenkins in South Africa [1]. Initially, the technology was used for cold deep recycling of asphalt pavements [2,3]. With groundwork laid, the interest in water foaming of bitumen spread across borders [4,5,6,7,8]. Researchers looked at how various modifiers affect the properties of bitumen after foaming [9,10] and evaluated the impact of different minerals, including waste materials, on the properties of recycled asphalt mixtures [11,12].

Sustainable technologies aimed at emission reductions have contributed to the development of asphalt mixtures produced at temperatures of about 120–140 °C. Traditional hot mixes are produced at about 170 °C, depending on the type of bitumen used [13,14]. With the emergence of Warm Mix Asphalt (WMA) technologies [15,16,17], water-foamed bitumen was found to be applicable to mixtures used in pavement upper layers [18,19]. Further development allowed lowering mix production temperatures even more, to about 90–110 °C (Half Warm Mix Asphalt, HWMA) [20,21,22]. At the same time, to ensure high quality of the HWMA material, researchers looked at the influence of various chemical compounds on the binder basic parameters, i.e., maximum expansion (ER) and half-life (HL) [9,23], and properties of foamed bitumen mixtures [24,25,26]. 

Compared with conventional technology, low production temperature of HWMA and WMA with and without foamed bitumen alleviates the negative impact of high temperature on the bitumen [12,13], reduces the rate of its ageing [27,28,29], and generates smaller changes in binder parameters and mixture properties [30,31,32]

Foamed bitumen binder is produced by subjecting the bitumen to the effect of pressurized water injected together with air. Rapid vaporization of water in the bitumen forms the bitumen foam [1,4,24]. Water and air affect the internal structure of the binder by initiating processes that change its properties after the foam decays and the bitumen regains its original consistency. Here, bitumen ageing is not due to temperature but rather to the action of pressurized water vapour and oxygen contained in the air. Due to the specificity of the foaming process and the fact that bitumen foam is primarily used in recycled base layers, this issue has not yet been widely investigated. 

Alterations of bitumen chemical composition and morphology due to ageing can be successfully observed using Atomic Force Microscopy (AFM) [33,34] and ATR spectroscopy. Such changes were demonstrated in the study reported by Iwański et al. [30]. Their findings were consistent with the test results presented by Dong et al. [35].

Bitumen ageing in conventional mixtures is not as important as in HWMA and WMA intended for pavement upper layers [13,14,30,36,37]. The use of foamed bitumen reduces the short-term ageing process to a very short period during its injection into the mineral mix at <100 °C. More frequent use of bituminous mixtures in the upper layers, in particular in the wearing course, makes it necessary to identify the effects of the bitumen foaming process on the standard and rheological characteristics of the binder. 

## 2. Materials and Research Methodology

### 2.1. Tested Materials

Bitumen 70/100 used in this work was produced at ORLEN S.A refinery in Płock. This bitumen grade is commonly used in the Central and Eastern European countries for cold recycled bases in pavements under traffic loads of 2.5 × 10^6^ < ESAL_100 kN_ < 7.3 × 10^6^ (ESAL—equivalent single axle load) [38]. Table 1 compiles the basic properties of this binder. 

Proper preparation of binder samples is critical for the quality of the test and uniformity of the results. The samples were shipped in 5 L containers directly from the refinery and then transferred to 1L steel cans in accordance with EN 12594 [43]. In the next step, the samples were pre-heated to the softening point of the binder plus 100 °C and stirred at the rate of 150 rpm for 30 s, and then 600 rpm for 270 s. The 250 g test samples were placed in the heated vacuum chamber to remove air bubbles. The quality of the test samples was assessed through macroscopic examination. 

### 2.2. Experimental Program

Tests on bitumen 70/100 before and after foaming involved three stages. First, the foaming parameters were evaluated. Then the basic, rheological and low-temperature properties of the binder, as well as the effect of foaming on these properties were determined. 

Basic parameters of bitumen foam are used to determine the optimal amount of foaming water. Optimization result is typically the relationship between the amount of water used and two foaming parameters: *ER_m_* (-) and HL (s). The Technical Guidelines [5] describe the test methodology in detail. Since the measurement accuracy can be affected by the researcher, a laser-based method was used to measure bitumen foam changes over time.

An original digital measuring device was developed at the Kielce University of Technology [44] to increase the accuracy of the results. The device was designed based on literature data [45,46] pointing to a strong dependence of the measurement results on the researcher’s experience.

**Figure 1:**
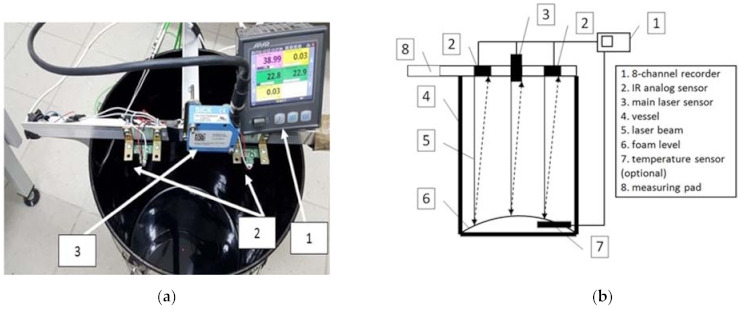
Design of the original measuring pad for measuring bitumen foaming parameters (1—recorder, 2—analog IR sensors of distance measurement, 3—laser sensor): (**a**) photograph of the device; (**b**) schematic diagram [44].

The device employs a highly accurate laser sensor, similar to that used by Newcomb et al. [47]. The non-contact measurement of displacement was based on the principle of optical triangulation. A laser diode illuminates a point on the surface to be measured. The light reflected from this point is directed to the photosensitive matrix, where it is processed in real time. The declared accuracy of the distance measurement for the 50 mm range is ±15 µm. A photo of the device and digital recorder, as well as the schematic diagram of the device are shown in Figure 1.

The binder was foamed at 160 °C, 6.0 kPa water pressure and 5.5 kPa air pressure, with the foaming water content of 2.5% by weight of the binder. The bitumen foam was taken directly from the foaming nozzle into glass containers and left to cool to room temperature. In order to prepare test specimens, the foamed binder was reheated as per EN 12594 [43] requirements.

The foaming characteristics quantified during the first stage of the study included:

- maximum expansion ratio, *ER_m_* [1,5],
- half-life, HL [1,5],
- foam index, *FI* [1].

As recommended by Jenkins [1], the quality of the foamed bitumen was assessed using the foam index (*FI*), which is measured in seconds and calculated using the following formula:

(1) $$FI=\frac{-\mathrm{HL}}{ln2}\cdot \left(4-ER_{m}-4ln\cdot \left(\frac{4}{ER_{m}}\right)\right)+\left(\frac{1+C}{2C}\right)\cdot ER_{m}\cdot t_{s}\;\left(s\right)$$

where:

*C*—correction factor (ratio of measured and actual *ER*, *ER*_m_*/ER*_a_),
*HL*—half-life (s),
*t_s_*—discharge time (s),
*ER_m_*—measured expansion ratio (immediately after foaming),
*ER_a_*—actual expansion ratio.

Physical properties of the foam were evaluated at the Foaming Water Content (FWC) of 1.5%, 2.0%, 2.5%, 3.0%, 3.5%, and 4.0% in the WLB-10S laboratory plant. The test conditions [5] were as follows:

- temperature of bitumen: 155 °C,
- temperature of water: 20 °C,
- water flow: 100 g/s,
- foaming time: 5 s,
- air pressure: 500 kPa,
- water pressure: 600 kPa.

Optimum values of ER and HL were determined using a mathematical formula that represents the relationship between the foaming parameters and the water content. The optimum FWC should ensure the highest expansion and the longest half-life. 

The second stage of the testing program involved determining the pre and post foaming basic properties of the bitumen:

- penetration at 25 °C (*Pen,* EN 1426:2015-08) [39], which is a measure of bitumen consistency,
- softening point (*T_R&B_*, EN 1427:2015-08) [40],
- Fraass breaking point (*T_Fraass_*, EN 12593:2015-08) [41],
- dynamic viscosity at 60 °C, 90 °C, and 135 °C (EN 13302-2018) [42].

The temperature range of binder plasticity (*PR*), which depends on its softening point (*T_R&B_*) and breaking point (*T_Fraass_*), was determined from:

(2) $$PR=T_{R\&B}-T_{\mathrm{Fraass}}\;\left({}^{\circ}C\right)$$

In the final stage, the rheological and low-temperature properties of the binder before and after foaming were determined through:

- rheological tests: dynamic modulus, dynamic viscosity, and phase angle at temperatures 13 °C, 25 °C, and 40 °C and loading time (0.1–10 Hz) to EN 14770 [48], and
- low-temperature tests to EN 14771 [49].
- The binders before BBR testing were adequately subjected to short-term ageing in the rolling thin-film oven (RTFOT, 2011, Matest, Treviolo, Italy).

**Figure 2:**
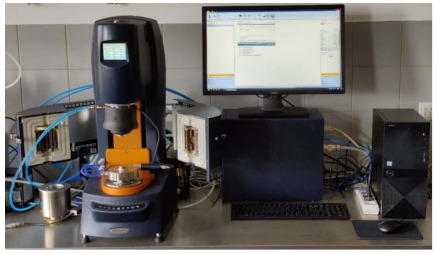
Discovery Hybrid Rheometer DHR-2.

The rheological tests for linear viscoelasticity of bitumen 70/100 were performed before and after foaming using a DHR-2 dynamic shear rheometer ((Discovery Hybrid Rheometer, 2019, TA Instruments, New Castle, DE, USA) shown in Figure 2.

The linear viscoelasticity region was determined according to the SHRP-A-370 report [50]. The maximum strain amplitude of the dynamic modulus |G*| for temperatures +13, 25, 40, and 60 °C was set at 0.1%, which corresponds to a stress of <50 Pa. The purpose of testing the dynamic modulus at different temperatures was to construct a master curve model based on the Time Temperature Superposition Principle (TTSP). The modified Huet-Sayegh model (2S2P1D) [51] was used. The same model had previously been applied by the authors to assess the rheology of the bitumen recovered from pavement layers [52].

**Figure 3:**
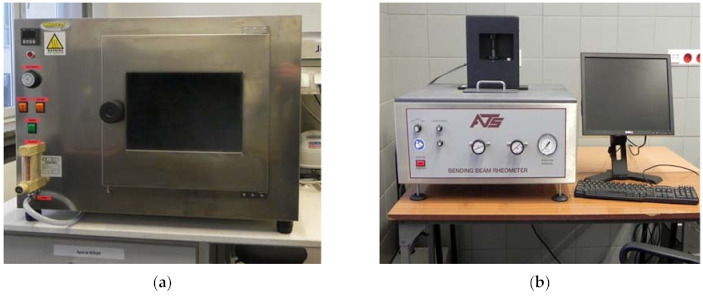
Test setup for determining low-temperature stiffness modulus of bitumen; RTFOT (**a**), BBR (**b**).

Another important step in investigating bitumen rheology was the evaluation of low temperature stiffness of bitumen samples using the Bending Beam Rheometer (BBR2S Bending Beam Rheometer, Applied Test Systems, Butler, PA, USA) method was used [44,53]. The testing setup used is shown in Figure 3.

Analysis of the bitumen low temperature response allowed the evaluation of low temperature relaxation modulus corresponding to the instantaneous stiffness modulus.

## 3. Results

### 3.1. Properties of Water-Foamed Bitumen 70/100

**Figure 4:**
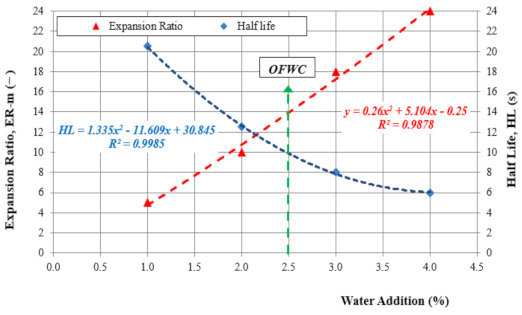
Optimum water content for foaming bitumen 70/100.

The suitability of bitumen 70/100 for foaming was assessed in accordance with the methodology used by the authors of [54]. The optimum amount of water was determined in accordance with the guidelines [5]. Nine determinations of the given foaming parameter were made. The results are shown in Figure 4.

The relationship between *ER_m_* and HL was the basis for determining the optimum FWC = 2% by weight of the binder for which *ER_m_* = 12 and HL = 12 s and *FI* = 122.

### 3.2. Basic Properties of Bitumen

The present study aimed to evaluate the influence of the foaming process on the basic properties of bitumen 70/100. The plan of the experiment included determining penetration at 25 °C (*Pen*) as per EN 1426 [39] (10 replicates), the softening point by the “ring and ball” method (*T_PiK_*) as per EN 1427 [40] (6 replicates), and the Fraass breaking point (*T_Fraass_*) as per EN 12593 [41] (4 replicates) before and after foaming. Table 2 shows the mean results of the penetration, softening point and breaking point determinations. Since the analysis was to evaluate the effect of the foaming process on these parameters, the results in individual series were designated as 70/100 for the non-foamed binder and as F 70/100 for the foamed binder.

c the softening point of 46.8 °C and the breaking point of −17.8 °C, and met the requirements of EN 12591 in the above range. It follows from the mean values that after foaming the penetration value decreased by an average of 2.2 (0.1 mm), the softening point decreased by 0.5 °C, and the breaking point increased by 0.3 °C. Standard deviation for penetration and softening point also increased after foaming.

The statistical significance of the foaming process for the bitumen 70/100 basic properties was assessed using a one-way analysis of variance ANOVA, the results of which are presented in Table 3 [55,56].

The results of statistical analysis showed a significant influence of foaming on the softening point. The changes in penetration and breaking point values were not significant.

**Figure 5:**
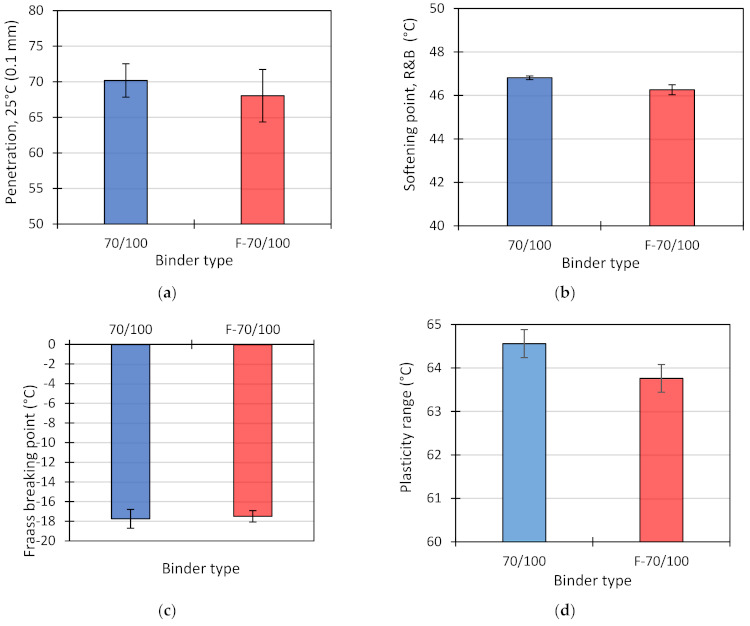
Properties of bitumen 70/100 before and after foaming: penetration at 25 °C (**a**); softening point (**b**); Fraass breaking point (**c**), plasticity range (**d**); error bars represent standard deviation.

Figure 5 shows the mean values of the basic properties of bitumen 70/100 before and after foaming, together with standard deviation. The data in Figure 5 confirm the ANOVA results in that the influence of the foaming process on the values of penetration and breaking point is not statistically significant. Despite the demonstrated statistical significance of foaming for the softening point, the difference between its values before and after foaming is marginal.

The test results for the basic properties of bitumen 70/100 (penetration at 25 °C, softening point (ring and ball) and breaking point) before and after foaming allow the following conclusions:

- non-foamed binder 70/100 meets the requirements set forth in EN 12591; after foaming, the penetration value of the binder was lower (68.0 (0.1 mm); min = 70.0 (0.1 mm));
- the foaming process had a statistically significant effect only on the softening point, which dropped from 46.8 to 46.3 °C;
- foaming had non-significant impact on penetration and Fraass breaking point,
- the plasticity range of the binder after foaming decreased slightly (from 64.6 to 63.7) as a result of a decrease in the softening point and a slight increase in the Fraass breaking point.

Dynamic viscosity changes were compared as a function of temperature. The tests were performed at four temperatures: 60, 90, 135 and 150 °C. The dynamic viscosity test was performed at a constant rotational speed of s^−1^. Three bitumen samples were tested. The range of the results did not exceed 5% [42]. Viscosity variations were determined by fitting the objective function, which was the Arrhenius-Guzman equation [57] in the form:

(3) $$\eta =A\cdot e^{\left(\frac{B}{T}\right)}$$

where:

*A*, *B*—material constants of the fluid,
*T*—temperature, °C,
*η*—dynamic viscosity, Pas.

**Figure 6:**
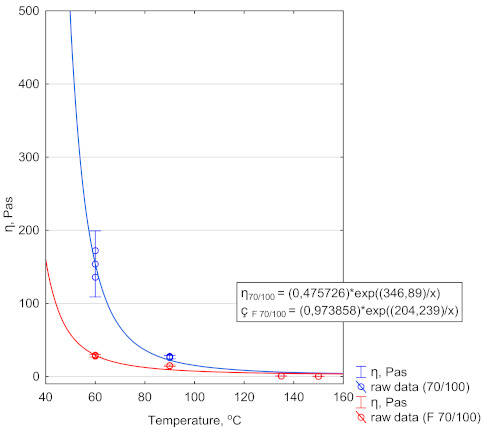
Dynamic viscosity of bitumen 70/100 and bitumen F 70/100 (after foaming). Error bars demonstrate the confidence interval.

Figure 6 shows the fitting result and the plot of the function.

From the data in Figure 6 it follows that the largest difference between dynamic viscosities of 70/100 and F 70/100 occurred at the temperature lower than 100 °C. The rapid drop in the value of dynamic viscosity of bitumen F 70/100 was related to its more porous nature. The air bubbles remaining after the foaming process disturbed the structure of bitumen F 70/100 at the lower temperature. The dynamic viscosity fell due to the presence of dispersed air spheres. The variation of coefficient B in model (3) confirms this observation. The B coefficient is directly related to the viscous flow activation energy. It expresses the molar energy necessary to overcome the intermolecular forces that inhibit the sliding of the layers with fluid flow. Thus, the low value of factor B of bitumen F 70/100 compared with 70/100 explains the rapid decrease in dynamic viscosity. At temperature higher than 100 °C, the viscosity of bitumen F 70/100 was low enough to eliminate the impact of air voids on dynamic viscosity which reached a similar level for both bitumen types.

### 3.3. Rheological and Low-Temperature Tests

Rheological tests were performed on the reference bitumen before foaming (70/100) and after foaming (F-70/100). Bitumen F-70/100 was tested at the half-life (HL) of 180–240 min.

The low temperature stiffness modulus is an important indicator used for the assessment of bitumen rheology.

Analysis of bitumen low temperature behaviour included an estimation of the low temperature relaxation modulus corresponding to the instantaneous stiffness modulus.

The following rheological tests of the reference bitumen (70/100) and foamed bitumen (F 70/100) were performed:

- BBR stiffness modulus in accordance with EN 14771 [49],
- dynamic modulus test |G*| at temperatures +13, 25, 40, and 60 °C and the frequency range of 0.1 to 10 Hz in accordance with EN 14770 [48].

**Figure 7:**
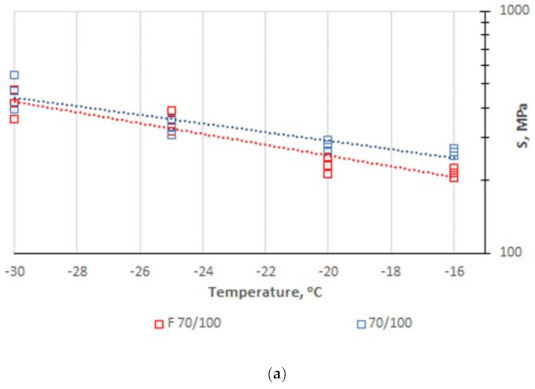

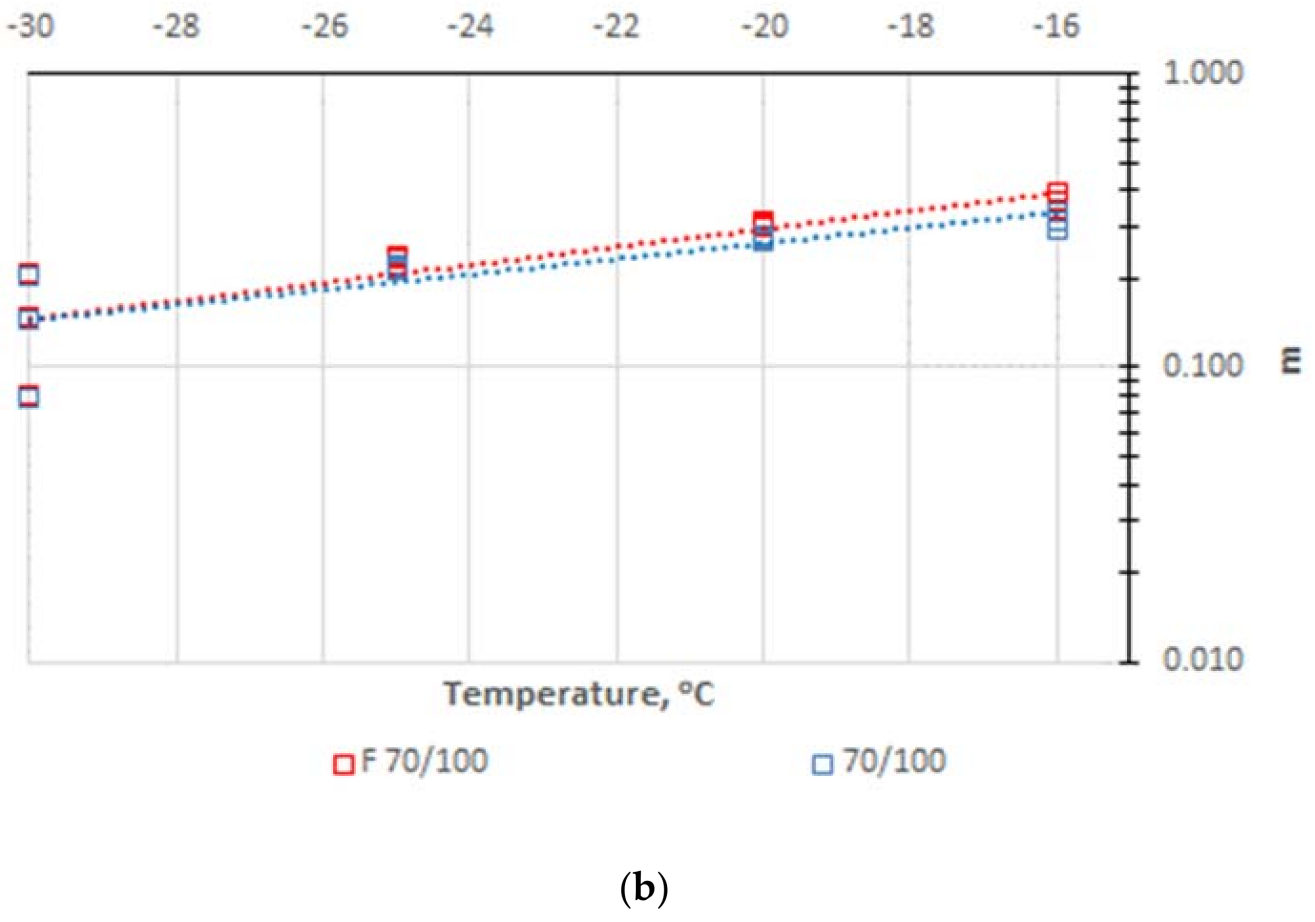
Creep parameter change at low temperature (BBR); (**a**) stiffness at 60 s, (**b**) coefficient m.

Four measurements were made at each of the test temperatures (−16, −20, −25, and −30 °C) [53]. A graphical presentation of the results is given in Figure 7.

The low-temperature properties assessment was based on parameters S(60) and m(60) obtained from three replications of the samples prepared separately for each temperature [49]. Parameter S(60) represents the temperature at which the bitumen sample reached the value of 300 MPa. The accompanying parameter m(60) represents a slope of the curve. The highest temperature value from the S(60) and m(60) determination is the critical temperature of the binder. Table 4 summarizes the values of low-temperature properties.

Since the critical temperature in the case of bitumen F 70/100 (−19.8 °C) is lower than that of the reference binder (−17.9 °C), and the stress measured at −16 °C as per EN 14023 [58] is also lower, the use of F 70/100 in a cold-recycled mix ensures its increased and beneficial compliance at low temperatures. Maintaining the increased flexibility in the low temperature range is likely to minimize the occurrence of low temperature cracking in the structural layers made of recycled mixtures. This phenomenon has the greatest impact on mix rheology at low temperatures. Bitumen F 70/100 will thus have a beneficial effect on the relaxation rate of thermally induced stresses [57].

Bitumen 70/100 was subjected to a series of rheological tests for linear viscoelasticity before and after foaming using a dynamic shear rheometer. Dynamic tests were performed to determine the characteristic master curves, which were in fact a relaxation function model. As it is not possible to determine the dynamic modulus using one measuring system, the tests were performed for one frequency range at several temperature levels. Using the superposition principle [51,59], master curves were constructed (2S2P1D model) for both bitumen types. The BBR tests aimed to determine the bitumen maximum stiffness at the load approaching zero (high frequency). The value estimated for the solution region <1 Pa was adopted as the value of the bitumen dynamic modulus as time tends to infinity.

The model consists of two springs:

- G_∞_ (equivalent of the static modulus/the loading time tends to infinity),
- G_0_ (instantaneous modulus/the loading time tends to zero),
- two parabolic dashpots: h and k,
- a linear dashpot β (defined by zero shear viscosity η_0_),
- t—loading time,
- h—exponent changing from 0 to 1 (h = 0 elastic behaviour, h = 1 viscous behaviour).

After the transformation of the 2S2P1D model in the frequency domain, the function describing time-dependent variation in |G*| has the form:

(4) $$G^{*}\left(\omega \right)=G_{0}+\frac{G_{g}-G_{0}}{1+\alpha {\left(i\omega \tau \right)}^{-k}+{\left(i\omega \tau \right)}^{-h}+\alpha {\left(i\omega \beta \tau \right)}^{-1}}$$

where:

$G^{*}\left(\omega \right)$—dynamic modulus in the frequency domain,
k and h—exponents 0 < k < h < 1 h changing from 0 to 1 (h = 0 elastic response, h = 1 viscous response),
α, β—constants,
τ—characteristic time,

Using the relationship below:

(5) $$\eta_{0}=\left(G_{g}-G_{0}\right)\beta \tau$$

where $\eta_{0}$—zero shear viscosity, it is possible to determine dynamic viscosity for long-term loading. As it has an influence on the increase in plastic deformation rate in asphalt concrete, this parameter can be helpful in determining asphalt mixture susceptibility to permanent deformation (rutting).

The estimation of the model parameters required the use of the nonlinear least squares method to minimize the objective function at established initial values. A complex block script was prepared in MathCad using a solver with the implemented quasi-Newton method [60].

In order to estimate the seven parameters of the model, at least eight determinations of the dynamic modulus |G*| at different frequencies are required. The mechanical parameters of the 2S2P1D model are independent of temperature, unlike those in the widely used Burger model. The characteristic time can only be described by temperature, which is the correct solution when the time-temperature superposition principle (TTSP) is used in the model. This allows building master curves to describe changes in complex modulus values with time. The TTSP was determined from the modified equation:

(6) $$\tau =\tau_{0}e^{\left(A_{0}+T\cdot A_{1}\right)}$$

where:

T—test temperature,
$\tau_{0}$—initial characteristic time,
A_0_, A_1_—parameters of the model.

This method of defining the time-temperature superposition (6) is characterized by a compromise between the quality of parameter estimation and the number of its parameters. The last step in the approximation of 2S2P1D model parameters is assessing the fit of the model to the values obtained. Two qualitative measures were used: the coefficient of determination R^2^ and the mean normalized error, MNE [61,62]. Since these measures are not correlated, the high quality of the model must rely on high R^2^ and low MNE.

**Figure 8:**
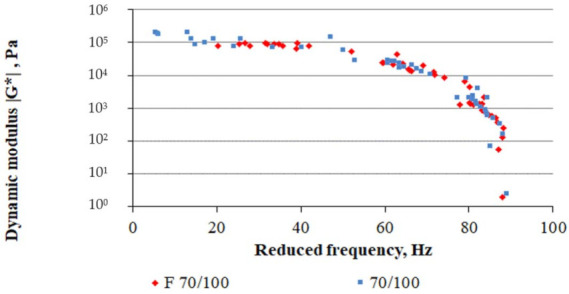
Black curve for bitumen 70/100 before and after foaming.

Model parameter estimation was based on the value of the dynamic modulus |G*| and the phase angle δ. The overall results are presented using the Black chart. Figure 8 compares the rheological nature of the bitumens before and after foaming.

**Figure 9:**
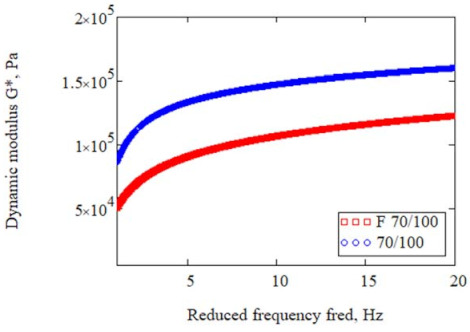
Master curves of bitumen 70/100 and bitumen F 70/100 at 40 °C based on the 2S2P1D model.

Analysis of the results in Figure 8 shows that the foaming process changed the rheological character of the binder. Reference bitumen 70/100 had much higher stiffness in the temperature range of 25 °C, as indicated by the small phase angle results. The phase angle values for bitumen F 70/100 are shifted to the range of higher values, which suggests that the viscous component G″ of the dynamic modulus |G*| will dominate in this bitumen. As a result, the bitumen will be more susceptible to the creep effect. However, when used as a dispersed phase in a cement-based mixture, it will reduce the stiffness caused by the presence of the hydraulic binder. In the range of high phase angles, the difference between the foamed bitumen F 70/100 and the reference bitumen 70/100 was negligible. The rheological nature of both bitumens was compared by plotting master curves for 40 °C using the 2S2P1D model, as shown in Figure 9.

The plot of the master curve at 40 °C (Figure 9) indicates that compared with F 70/100, the reference bitumen (70/100) obtains higher values of the dynamic modulus |G*| and thus higher stiffness over the entire frequency range. Also, the shape of the 70/100 master curve suggests that the bitumen reaches its elastic state much faster. Interpretation of the master curve was based on the comparison of its parameters, which are not temperature-dependent. The 2S2P1D model parameters for the reference and foamed bitumens are tabulated in Table 5.

**Figure 10:**
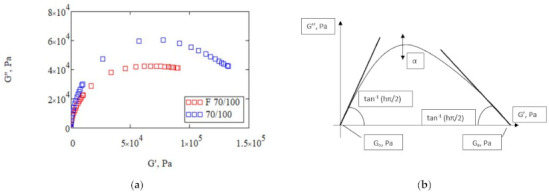
Cole-Cole plot: (**a**) results of bitumen 70/100 before and after foaming; (**b**) graphical representation of the 2S2P1D model.

Analysis of the 2S2P1D model (Table 5) shows that the phase angle δ for the reference bitumen is higher than for the foamed binder. Its value suggests a higher level of |G*| in the reference bitumen as confirmed by the plot of the master curve in Figure 9. The zero shear viscosity η_0_ for bitumen 70/100 is nearly twice as high as that of F 70/100. This indicates lower resistance of F 70/100 to permanent deformation, which is related to the amount of air bubbles trapped in the bitumen phase contributing to the viscous character of the binder. Sensitivity of the binder to the effects of a long-term loading is also indicated by the *h* factor, which for F 70/100 is twice that of the reference bitumen. In fact, the deformation of foamed bitumen under long-term loads will occur nearly twice as fast as in the reference bitumen. Parameters *h* and *k* describe the slope of the curve in the Cole-Cole diagram (Figure 10).

Each of the parameters of the 2S2P1D model plays a specific role in shaping the Cole-Cole plot (Figure 10b):

- k controlled the slope at high values of G″ in the Cole-Cole diagram,
- h controlled the slope at low values of G″ in the Cole-Cole diagram,
- δ controlled the slope at the low temperatures/high frequencies in the |G*| master curve and the height of the pinnacle point of the Cole-Cole diagram,
- β controlled the slope at the high temperatures/low frequencies of the |G*| master curve. It is also correlated with the zero shear viscosity η_0_.

The master curve projected on the G′-G″ plane (Figure 10a) shows that 70/100 is markedly stiffer compared to F 70/100. Also, the k value for the reference bitumen (k = 0.13) is approximately 30% higher than that of the foamed bitumen (k = 0.1). Accordingly, the h parameter representing the bitumen viscous nature is lower in F 70/100 than in 70/100. This demonstrates that the reference bitumen (70/100) will have a more elastic character than bitumen F 70/100. It must be emphasized that the presence of entrapped air reduced zero shear viscosity η_0_ in F70/100, which suggests that the mixture will show higher susceptibility under long-term loading.

Nevertheless, the dominance of G″ over G′ will probably improve the fatigue life of the asphalt mixture with F 70/100 compared to that produced with bitumen 70/100 [63,64]. The lower value of δ = 1.7 of F 70/100 relative to 70/100 (δ = 2.01) suggests a better stress relaxation capability of F 70/100, which is important in terms of fatigue life of the asphalt mixture.

## 4. Conclusions

Foaming has a considerable effect on the basic parameters and rheology of bitumen 70/100, as demonstrated in this study. Conclusions drawn from the tests and observations are as follows:

- Water-foaming changes basic characteristics of bitumen such as penetration at 25 °C, softening point and breaking point. In the case of the softening point, this effect is more pronounced and statistically significant.
- Water-foaming reduced the critical temperature of the bitumen. The stress read at −16 °C as per EN 14023 [58] was lower for foamed bitumen F 70/100 than for the reference bitumen 70/100.
- The critical temperature was −19.8 °C in F 70/100 and −17.9 °C in 70/100. Therefore, the application of bitumen F 70/100 will ensure that a cold recycled cement-based mixture in which it is incorporated will have improved and more beneficial compliance in the range of low temperatures.
- The foaming process changed the rheological character of the bitumen under test. The reference bitumen 70/100 exhibited considerably higher stiffness at 25 °C range, as demonstrated by small values of the phase angle. Higher values of the phase angle for F 70/100 indicate that the viscous component G″ of the dynamic modulus |G*| will dominate. As a result, this bitumen will be more *susceptible to creep*.
- The plot of the master curve at 40 °C indicates that, compared with the foamed bitumen, the reference bitumen attains higher values of the dynamic modulus |G*|, hence the higher stiffness over the entire frequency range. The shape of the bitumen 70/100 curve suggests that this bitumen type attains elastic state much faster than F 70/100.

In summary, foaming changes the properties of bitumen and the parameters of cold and warm asphalt mixtures. However, the analysis of foamed bitumen properties and the results of numerous field and laboratory experiments indicate that this change does not affect the main mechanical characteristics of the mixture but contributes to its high quality [11,21].

## Figures and Tables

**Table 1:** Basic properties of 70/100 bitumen.

Property	Test Method	Unit of Measure	Result
Penetration at 25 °C	EN 1426 [39]	0.1 mm	70
Softening point *T_R&B_*	EN 1427 [40]	°C	47
Fraass breaking point	EN 12593 [41]	°C	−18
Dynamic viscosity at:	EN 13302:2011 [42]	Pa∙s	
60 °C			154
90 °C			27
135 °C			0.9

**Table 2:** Determined properties of bitumen binder 70/100 before and after foaming.

Binder Type	*Pen* (0.1 mm)		*T_R&B_* (°C)		*T_Fraass_* (°C)	
	Mean	SD	Mean	SD	Mean	SD
70/100	70.2	2.346	46.8	0.085	−17.8	0.957
F 70/100	68.0	3.690	46.3	0.230	−17.5	0.577

**Table 3:** One-way analysis of variance. Significance of foaming effect (independent variable: *Binder*) on the properties of bitumen 70/100: *Pen*, *T_PiK_*, *T_Fraass_*.

variable: *Pen*	Repeatability Error	*p (α = 0.05)*
Binder	9.600	0.105
variable: *T_R&B_*	Repeatability	*p (α = 0.05)*
Binder	0.030	<0.001
variable: *T_Fraass_*	Repeatability	*p (α = 0.05)*
Binder	0.625	0.670

**Table 4:** Stiffness modulus S, parameter m, and critical temperature.

Bitumen	S(60) = 300 (MPa) T(S) 60 (°C)	m(60) = 0.3 T(m) 60 (°C)	S(T)_−16_ (MPa)
70/100	−19.2	−17.9	262
F 70/100	−21.3	−19.8	215

**Table 5:** 2S2P1D model parameters for the bitumen (70/100, F 70/100) under analysis.

Bitumen	2S2P1D Model Parameters								
	δ	k	h	β/η_0_	G_o_/G_∞_	τ	A_0_/A_1_	R^2^	RMSE
	(-)	(-)	(-)	(-)/Pas	(Pa)	(s)	(-)	(-)	(%)
F 70/100	1.7	0.13	0.26	172.3/1 × 10^7^	3.5210^−5^/8.93 × 10^5^	0.065	−0.201/2.5 × 10^−3^	0.97	9.2
70/100	2.01	0.28	0.56	12.6/2.97 × 10^7^	0.027/2.37 × 10^6^	0.1	−0.22/0.158	0.99	14.0

## Data Availability

Data available on request from the corresponding author.
